# Potentially inappropriate medication use among geriatric patients in primary care setting: A cross-sectional study using the Beers, STOPP, FORTA and MAI criteria

**DOI:** 10.1371/journal.pone.0218174

**Published:** 2019-06-13

**Authors:** Abdelmoneim Awad, Olivia Hanna

**Affiliations:** Department of Pharmacy Practice, Faculty of Pharmacy, Kuwait University, Kuwait, Kuwait; Nederlands Instituut voor Onderzoek van de Gezondheidszorg, NETHERLANDS

## Abstract

Inappropriate prescribing is a risk factor for adverse drug reactions and hospitalizations in the elderly and places a considerable burden on the healthcare system. Hence, it is imperative to identify irrational prescribing and implement interventions to improve prescribing appropriateness in geriatric clinical practice. This study aimed to determine: (i) the prevalence of potentially inappropriate medications (PIMs) according to Beers STOPP, FORTA, and the Medication Appropriateness Index (MAI) criteria; (ii) the prevalence of potential prescribing omissions (PPOs) according to START criteria; and (iii) the predictors for PIMs and PPOs. A cross-sectional study was performed among elderly outpatients of 10 primary healthcare centers with specialized geriatric clinics in Kuwait. Four-hundred and seventy-eight patients were selected randomly, 420 (87.9%) agreed to participate. Data about chronic diseases and prescribed medications were obtained from the physicians by accessing the patients’ medical records. Descriptive and multivariable logistic regression were used for data analysis. A total of 2645 medications were prescribed to all patients; mean (SD) number of medicines per patient was 6.3 (3.0). PIMs were present in 53.1%, 55.7%, and 44.3% of respondents, according to Beers, STOPP, and FORTA criteria, respectively. Almost 74% of respondents had one or more inappropriate ratings among their medications in the MAI criteria. According to START criteria, 19.8% of patients had at least one PPO. Respondents taking ≥ 5 medications were found to be using more PIMs according to Beers (OR: 6.3), STOPP (OR: 3.3), FORTA (OR: 6.0) and MAI (OR: 3.9) criteria in comparison to those taking ≤ 4 medications (p<0.001). The MAI revealed a significantly higher number of medications with inappropriate ratings compared to the Beers, STOPP and FORTA criteria (p<0.001). Taking the MAI as reference standard, STOPP criteria had the highest sensitivity (68.6%) and measure of agreement (Kappa index = 0.40) to detect PIMs compared with Beers and FORTA criteria. Inappropriate prescribing is common among the elderly in the primary geriatric clinics. This necessitates further evaluation of its impact on clinical outcomes and warrants efforts to implement interventions to improve prescribing practice in these settings.

## Introduction

The world’s geriatric population continues to increase rapidly. The current statistics indicate that 8.5% of the world’s population are aged ≥ 65 years and is expected to increase to 17% by 2050 [1]. In Kuwait, the geriatric population represents 2.33% (96,600) of the estimated total population, and it is expected to increase to 4.41% and 17.9% by 2025 and 2050, respectively [2]. Given the increase in this population, there is an ever-greater need to improve their health, quality of life, and promote the optimal prescribing of medicines.

Appropriate prescribing in geriatric patients is a challenging and complex process due to several characteristics of ageing [3]. These include an increase in the prevalence of prescribing multiple medicines as incidence of multiple chronic diseases and degenerative conditions increases, and age-related physiological changes that affect the pharmacodynamics and pharmacokinetics profiles of medicines. Furthermore, there is paucity of literature reports regarding the use of medicines in geriatrics and the manufacturers do not include geriatric patients in the clinical trials prior to marketing. These factors make geriatric patients more prone to drug-related adverse events combined with drug-drug and/or drug-disease interactions, increased hospitalization and increased healthcare costs [3–5].

The concern regarding the impact of inappropriate prescribing among the elderly population has led to the conception of several strategies to deal with this common problem, among these is the detection of potentially inappropriate medications (PIMs). Screening tools with explicit criteria to detect various aspects of PIMs were developed to assist the healthcare providers in selecting safer therapy, and lessen the exposure of the elderly to PIMs. Two sets of tools have acquired international recognition: the American Geriatric Society Beers Criteria and Screening Tool of Older People’s Potentially Inappropriate Prescriptions criteria and Screening Tool to Alert Doctors to Right Treatment (STOPP/START) criteria [6, 7]. A recently introduced evidence-based tool is FORTA (Fit fOR The Aged) list [8]. Also, the Medication Appropriateness Index (MAI) as a reliable, valid, and standardized assessment tool with implicit criteria is used to evaluate medication use in geriatrics [9–11]. The use of these criteria in epidemiological studies to address quality of prescribing in geriatric patients, has proven to be useful, and provides significant information to improve the treatment policies in health services [6, 7, 12, 13]. Several studies were conducted to describe the prevalence of PIMs among geriatrics in various settings including, the outpatient, hospital, and home care setting in different countries worldwide, particularly in Western countries [4, 5, 12, 14–37]. Few studies were performed in the Middle-Eastern region in 3 countries, namely Saudi Arabia [38–40], Lebanon [41, 42], and Qatar [43].

Although the evaluation of appropriateness of prescribing in geriatric patients is mandatory, there is lack of data on prescribing patterns of PIMs among this population in the different healthcare settings in Kuwait. Hence, this study was designed to determine: (i) the prevalence of PIMs among outpatients in primary geriatric clinics according to Beers, STOPP, FORTA, and the MAI criteria; (ii) the prevalence of PPOs according to START criteria; and (iii) the demographic and clinical predictors for PIMs and PPOs. It was reported that explicit criteria have limited transferability across various countries; however, implicit criteria although not drug-specific are more readily transferable across countries. The disadvantage of the implicit criteria is that they are more time consuming in terms of application [9, 16, 31]. Therefore, it was considered interesting as a secondary objective to compare between these criteria in detecting PIMs with greater applicability to the Kuwaiti population.

## Materials and methods

### Study area

Kuwait is a Middle-Eastern country with an area of 17,820 km^2^ and an estimated population of 4.2 million people (2018 estimate) [2]. In Kuwait, the healthcare system comprises public and private sectors. The public sector consists of primary, secondary, and tertiary levels of healthcare delivery. Primary care is provided through healthcare centers (polyclinics) disseminated over the six governorates of Kuwait. Six general hospitals provide secondary care, while specialized hospitals and health centers deliver tertiary care. Physicians employed in these health facilities have various educational backgrounds and training from Kuwait, and other countries such as Egypt, India, United Kingdom, United States of America, and Canada.

### Study design and population

A descriptive, prospective, and cross-sectional study was performed in 10 primary healthcare centers (polyclinics) that provide geriatric care through specialized geriatric clinics. It was conducted during the period from January to September, 2016. The target population was geriatric patients, aged ≥ 65 years attending these healthcare centers. This study was approved by the Ministry of Health Ethical Committee, Kuwait.

The sample size was based on the assumption that the proportion of patients with at least one PIM is 50% due to the fact there are no previous similar studies from Kuwait. It was determined using the Raosoft sample size calculator using a margin of error of 5%, a confidence interval of 95%, and a target population size of 65,024 individuals aged ≥ 65 years [44]. The minimum sample size estimated was 382. Assuming a response of 80%, a larger sample size of 478 elderly patients were approached to be included in the study.

A list of the primary healthcare centers that provide geriatric care through specialized geriatric clinics at the time of the study was obtained from the Ministry of Health. It included 10 geriatric clinics distributed among five governorates (3 in Al-Farwaniyah, 2 in the Capital, 2 in Al-Ahmadi, 2 in Al-Jahra, and 1 in Hawalli). The total number of geriatric patients’ files at each healthcare center was obtained, and stratified random sampling was used to determine the number of patients that should be approached at each healthcare center. The patients who attended these healthcare centers were selected using systematic random sampling.

### Data collection form

The data collection form consisted of two sections. The first section included questions to retrieve information about patients’ demographic characteristics (age, gender, education level, residence area) and smoking habits. The second section retrieved information about the patients’ chronic diseases and prescribed medications.

### Data collection

Data were collected prospectively via face-to-face structured interviews of the respondents in the geriatric clinics using the first section of the data collection form. Patients who agreed to participate in the study were assured for confidentiality and gave written consent to participate in the study. The attending physicians were asked to provide all necessary information regarding the patients’ chronic diseases and prescribed medications through accessing the patients’ medical electronic and non-electronic records. As-needed medications for chronic disease were included. Over-the-counter (OTC) medicines and herbal supplements were excluded as they were not well documented for each patient. Also, patients were not asked about self-medication with OTC and herbal products since some patients might not have clear recollection. Topical (eye drops, intranasal sprays) and dermatological medicines were excluded.

### Evaluation of prescribing appropriateness

The collected data were thoroughly reviewed and analyzed independently by two clinical pharmacists who are the authors of the present study. The first author is a professor of clinical pharmacy with experience in geriatric pharmacotherapy and the second author had a professional doctorate degree of pharmacy [PharmD]). Discrepancies were discussed until a consensus was reached. The PIMs were identified according to three explicit criteria that focused on the drug or on the disease and one implicit criteria. The explicit criteria used in the present study were as follows:

1. 2015 Beers criteria by the American Geriatric Society [6]. PIMs in the Beers criteria includes the following categories: medications to be avoided in the elderly patients regardless of medical conditions; medications to be avoided in combination with specific diseases or syndromes; and medications that should be used with caution; medications that should be avoided or have their dose adjusted based on kidney function; and selected clinical important drug–drug interactions documented to be associated with harmful effects in older adults.
2. STOPP/START version 2 criteria [7]. STOPP criteria consists of a section related to indication of medication (drug prescribed without an evidence-based clinical indication; drug prescribed beyond the recommended duration, where treatment duration is well defined; and duplicate therapy), and other sections in which medications are arranged according to a physiological system accompanied by an explanation of why they are potentially inappropriate. START criteria encompasses medications arranged according to a physiological system that should be considered for people with certain conditions (PPOs).
3. 2014 FORTA list [45]. Its classification system includes 4 classes defined as follows: (1) *Class A (A-bsolutely)*: *“indispensable drug*, *clear-cut benefit in terms of efficacy/safety ratio*, *proven in elderly patients for a given indication”;* (2) *Class B (B-eneficial)*: *“drugs with proven or obvious efficacy in the elderly*, *but limited extent of effect and/or safety concerns”*; (3) *Class C (C-areful)*:*” drugs with questionable efficacy/safety profiles in the elderly*, *which should be avoided or omitted in the presence of too many drugs*, *absence of benefits or emerging side effects; explore alternatives”*; and (4) *Class D (D-on’t)*: *“avoid if at all possible in the elderly*, *omit first and use alternative substances”*.

The MAI includes 10 implicit criteria (indication, effectiveness, dosage, correct directions, practical directions, drug-drug interactions, drug-disease interactions, duplication, duration and expense). Each medication prescribed to the patient is rated by these weighted criteria, resulting in a score per drug and in a summed patient score [9–11]. In the present study, the criterion concerning the cost of medication was not considered since Kuwaiti patients were given their medications free of charge, while the non-Kuwaitis have to pay a minimum charge. A total of 2645 medications were rated by 9 weighted criteria, a score of 1 to 3 is given to each criterion if it is inappropriate. Hence, a weighted MAI score for each medication can range from 0 indicating no prescribing problems to 17 if all 9 criteria are rated as inappropriate. The MAI score per patient is calculated by summing-up the total MAI scores of all the medications used by the patient.

### Statistical analysis

Data were analyzed using the Statistical Package for Social Sciences (IBM SPSS Statistics for Windows, version 23, Armonk, NY: IBM Corp). The results were presented as percentages (95% confidence intervals; CI), means (standard deviation-SD), medians and range. Univariable logistic regression was performed to determine the association of demographic and clinical predictors (age, gender, education, residence area, number of diseases and number of medications) with the dependent variables (PIM use according to Beers, STOPP, FORTA, MAI, and START criteria). All variables with p ≤0.25 in the univariable analysis were included in the multiple logistic regression analysis to determine the factors that are independently associated with each dependent variable. Only the results of multivariable logistic analysis are reported showing odds ratio (OR) and 95% CI. For each model, response options for the dependent variables were categorized as follows: (1) being prescribed PIM according to Beers criteria (0 = no, 1 = yes); (2) being prescribed PIM according to STOPP criteria (0 = no, 1 = yes); (3) being prescribed PIM according to FORTA criteria (0 = no, 1 = yes); (4) being prescribed medication with one or more inappropriate ratings in the MAI criteria (0 = summated patient score 0, 1 = summated patient score ≥ 1); and (5) had PPO according to START criteria (0 = no, 1 = yes). The independent variables were categorized as follows: (1) age: 65–74 years, 75–84 years, and ≥85 years; (2) gender: males and females; (3) level of education: low (less than secondary school), intermediate (completed secondary school), and high (had diploma, or bachelor degree or postgraduate degree); (4) residence area (Al-Farwaniyah, Capital, Al-Ahmadi, Hawalli, and Al-Jahra); (5) number of chronic disease (1–2 diseases and ≥ 3 diseases); and (6) number of medications (1–4 medications and ≥ 5 medications). Specificity and sensitivity of the explicit criteria were assessed using a two-by-two contingency table, and concordance between implicit and explicit criteria was estimated using kappa statistics. Spearman rank correlation was also used to analyze the association between the dependent variables. The comparison of the number of PIMs between the four screening tools was carried out using one-way analysis of variance (ANOVA). The comparison of proportions of patients identified with PIMs between the four screening tools was computed using EpiCalc 2000 (Centers for Disease Control and Prevention, Atlanta, GA).

## Results

### Demographic and clinical characteristics of the study participants

A total of 478 Kuwaiti elderly patients were approached to be included in the study, 420 agreed to participate giving a response rate of 87.9%. Their mean (SD) age was 73.1 (5.8) years (median 72; range 65–96). Of the 420 respondents, 56.7% were females and 85.5% had low-intermediate education. Table (1) shows the demographic and clinical characteristics of the study participants.

**Table 1:** Demographic and clinical characteristics of respondents (n = 420).

**Demographic Characteristics**	**Number (%)**
**Age (Years)**	
65–74	267 (63.6)
75–84	134 (31.9)
≥ 85	19 (4.5)
Mean (SD)	73.1 (5.8)
**Gender**	
Male	182 (43.3)
Female	238 (56.7)
**Education Level**	
Low	294 (70.0)
Intermediate Education	65 (15.5)
High Education	61 (14.5)
**Residence**	
Al-Farwaniyah	152 (36.2)
Capital	117 (27.9)
Al-Ahmadi	71 (16.9)
Hawalli	59 (14.0)
Al-Jahra	21 (5.0)
**Clinical Characteristics**	**Number (%; 95% CI)**
**Types of chronic diseases**	
Hypertension	330 (78.6; 74.3–82.3)
Diabetes	272 (64.8; 60.0–69.3)
Dyslipidemia	227 (54.1; 49.2–58.9)
Asthma	59 (14.1; 11.0–17.8)
Osteoarthritis	56 (13.3; 10.3–17.01)
Osteoporosis	51 (12.1; [9.3–15.8)
Ischemic heart disease	50 (11.9; 9.0–15.5)
Irritable bowel syndrome	30 (7.1; 5.0–10.2)
Hypothyroidism	28 (6.7; 4.6–9.6)
Depression	18 (4.3; 2.6–6.8)
Peripheral neuropathy	17 (4.1; 2.5–6.5)
Hyperuricemia/Gouty Arthritis	15 (3.6; 2.1–6.0)
Chronic heart failure	12 (2.9; 1.6-, 5.1)
Chronic kidney disease	8 (1.9; 0.9–3.9)
Benign prostatic hyperplasia	8 (1.9; 0.9–3.9)
Rheumatoid arthritis	6 (1.4; 0.6–3.2)
Dementia	6 (1.4; 0.6–3.2)
Atrial fibrillation	5 (1.2; 0.4–2.9)
Psychiatric disorder	4 (1.0; 0.3–2.6)
Sinusitis	4 (1.0; 0.3–2.6)
Epilepsy	4 (1.0; 0.3–2.6)
Chronic obstructive pulmonary disease	3 (0.7; 0.2–2.3)
Parkinson’s disease	2 (0.5; 0.1–1.9)
**Number of Diseases**	
1–2	128 (30.5; 26.2–35.2)
≥ 3	292 (69.5; 64.8–73.9)
**Number of Medications**	
1–4	117 (27.9; 23.7–32.5)
≥ 5	303 (72.1; 67.6–76.33)

Four hundred and nine (97.4%; 95% CI: 95.2–98.6) of the study participants were currently non-smokers, of whom 51 (12.5%) were ex-smokers. The most common chronic diseases among the participants were hypertension (n = 330; 78.6%), diabetes (n = 272; 64.8%) and dyslipidemia (n = 227; 54.1%). The mean (SD) numbers of diseases and medications per patient were 3.4 (1.6) diseases (median 3; range 1–9) and 6.3 (3.0) medications (median 6; range 1–16), respectively. Table 2 shows the categories of drugs prescribed to the study participants.

**Table 2:** Categories of drugs prescribed to respondents (n = 2645).

Categories of drugs	Number (%; 95% CI)
Cardiovascular drugs	1150 (43.5; 41.6–45.4)
Antidiabetic drugs	576 (21.8; 20.2–23.4)
Gastrointestinal drugs	192 (7.3; 6.3–8.3)
Respiratory drugs	157 (5.9; 5.1–6.9)
Endocrine drugs	62 (2.3; 1.8–3.0)
Central nervous system drugs	64 (2.4; 1.9–3.1)
Analgesics and non-steroidal anti-inflammatory drugs	67 (2.5; 2.0–3.2)
Skeletal muscle relaxants	29 (1.1; 0.8–1.6)
Vitamins, minerals and dietary supplements	247 (9.3; 8.2–10.5)
Others (such as bisphosphonates, allopurinol, antihistamines, and disease modifying anti-rheumatic drugs)	101 (3.8; 3.1–4.6)

### Potentially inappropriate medications (PIMs) according to Beers criteria

Over one-half (n = 223; 53.1%; 95% CI: 48.2–57.9) of the study participants were prescribed at least one PIM according to Beers criteria. From these patients, 140 (62.8%; 95% CI: 56.0–69.1) received only one PIM, 43 (19.3%; 95% CI: 14.4–25.2) received two PIMs, 26 (11.6%; 95% CI: 7.9–16.8) received three PIMs, and 14 (6.3%; 95% CI: 3.6–10.5) received four or more PIMs. Among 2645 medicines prescribed, 365 (13.8%; 95% CI: 12.5–15.2) were classified as PIMs according to Beers criteria. Over three-fifths (62.7%; n = 229; 95% CI: 57.5–67.7) were categorized as medications that should be used with caution, 38.4% (n = 140; 95% CI:33.4–43.6) as medications to be avoided in geriatrics regardless of medical conditions, 4.7% (n = 17; 95% CI: 2.8–7.5) as potentially clinically important drug-drug interactions to be avoided, 1.1% (n = 4; 95% CI: 0.4–2.9) as medications to be avoided in combination with specific diseases or syndromes, and medications that should be avoided or have their dosage adjusted based on kidney function. The therapeutic classes of PIMs were cardiovascular medications (54.6%), central nervous system medications (15.6%), gastrointestinal medications (10.7%), skeletal muscle relaxants (8.2%), non-steroidal anti-inflammatory medications (6.6%), glibenclamide (3.3%), and first-generation antihistamines (0.8%).

### Potentially inappropriate medications (PIMs) according to STOPP criteria

Based on STOPP criteria, 234 patients (55.7%; 95% CI: 50.8–60.5) were prescribed at least one PIM. Of these 234 patients, 155 (66.2%; 95% CI: 59.7–77.2) used only one PIM, 51 (21.8; 95% CI: 16.8–27.8) used two PIMs, 20 (8.6%; 95% CI: 5.4–13.1) used three PIMs, and 8 (3.4; 95% CI:1.6–6.9) used four or more PIMs. Among 2645 medicines prescribed, 351 (13.3; 95% CI: 12.0–14.6) were classified as PIMs based on STOPP criteria. The highest prevalence of PIMs (52.6%) was in relation to medications prescribed without an evidence-based clinical indication and medications prescribed beyond the recommended duration, and duplicate therapy. The remaining PIMs (47.4%) were related to medications whose primary effects are on the cardiovascular, endocrine, central nervous, musculoskeletal, and gastrointestinal systems.

### Potentially inappropriate medications (PIMs) according to FORTA list

Over two-fifths (n = 186; 44.3%; 95% CI: 39.5–49.2) of the patients were prescribed PIMs classified as C (n = 63; 33.9%; 95% CI: 27.2–41.2) or D (n = 102; 54.8%; 95% CI: 47.4–62.1) or C and D (n = 21; 11.3%; 95% CI: 7.3–16.9) medications based on FORTA list. From these patients, 137 (73.6%; 95% CI: 66.6–79.8) received only one PIM, 39 (21.0%; 95% CI: 15.5–27.7) received two PIMs, 7 (3.8%; 95% CI: 1.7–7.8) received three PIMs, and 3 (1.6%; 95% CI: 0.4–5.0) received four or more PIMs. Among 2645 medicines prescribed, 252 (9.5%; 95%CI: 8.5–10.7) were classified as C or D medications based on FORTA list. Of these medications, 97 (38.5%; 95% CI: 32.5–44.8) and 155 (61.5%; 95% CI: 55.2–67.5) were classified as C and D medications, respectively. The most common class C medications prescribed were attributed to cardiovascular (isosorbide mononitrate, amiodarone, moxonidine, and dabigatran), gastrointestinal (ranitidine), central nervous (mirtazapine, carbamazepine, pregabalin, gabapentin, and quetiapine), and endocrine (glimepride, repaglinide, and pioglitazone) systems. The most common class D medications prescribed were attributed to glibenclamide, verapamil, non-steroidal anti-inflammatory drugs, and benzodiazepines.

### Potentially inappropriate medications according to Medication Appropriateness Index (MAI)

A total of 2645 medications were prescribed to all patients. Almost three-quarters (n = 1969; 74.0%; 95% CI: 72.7–76.1) of these medicines were considered to be appropriate. However, 676 (25.6%; 95% CI:23.9–27.3) medications had one or more inappropriate ratings in the 9 criteria of the MAI. Four hundred and fifty-two out of 676 medications (66.9%; 95% CI: 63.2–70.4) met inappropriate ratings in 1–3 MAI criteria and 224 (33.1%; 95% CI: 29.6–36.9) met inappropriate ratings in ≥ 4 MAI criteria. The mean (SD) MAI score per drug was 1.0 (1.9) (median 0.0; range 0–9). Three hundred and nine (73.6%; 95% CI: 69.0–77.7) respondents had one or more inappropriate ratings among their prescribed medications. The mean (SD) MAI score per patient was 5.8 (5.8) (median 5; range 0–46). The criteria with the highest inappropriate percentages were effectiveness, duration of therapy, indication, drug-drug interaction and correct dosage. Table 3 shows the distribution of inappropriate prescribing for each MAI criterion.

**Table 3:** Distribution of inappropriate prescribing for each criterion of the MAI.

MAI Criteria	Drugs with an inappropriate MAI criterion (n = 2645)		Patients with an inappropriate prescribed medications (n = 420)	
	Number	Percentage (%; 95%CI)	Number	Percentage (%)
Indication	164	6.2 (5.3–7.2)	140	33.3 (28.9–38.1)
Effectiveness	357	13.5 (12.2–14.9)	233	55.5 (50.6–60.3)
Correct Dosage	152	5.8 (4.9–6.7)	133	31.7 (27.3–36.4)
Correct Directions	25	1.0 (0.6–1.4)	20	4.8 (3.0–7.4)
Practical Directions	19	0.7 (0.5–1.1)	16	3.8 (2.3–6.2)
Drug-Drug Interactions	155	5.9 (5.0–6.5)	54	12.9 (9.9–16.5)
Drug-Disease/Condition Interaction	21	0.8 (0.5–1.2)	19	4.5 (2.8–7.1)
Duplication	51	1.9 (1.5–2.6)	20	4.8 (3.0–7.4)
Duration of Therapy	169	6.4 (5.5–7.4)	134	31.9 (27.5–36.6)

### Potentially prescribing omissions (PPOs) according to START criteria

According to START criteria, 83 (19.8%; 95% CI: 16.1–24.0) of the study population had at least one PPO. Out of these, 48 (57.8%; 95% CI: 46.5–68.4) had one PPO, 22 (26.5; 95% CI: 17.7–37.5) had two PPOs, 8 (9.6%; 95% CI: 4.6–18.6) had three PPOs, and 5 (6.0%; 95% CI: 2.2–14.1) had four PPOs. The PPOs among these patients were bone anti-resorptive or anabolic therapy (e.g. bisphosphonate, teriparatide, denosumab) in patients with documented osteoporosis (39.8%), vitamin D and/ or calcium supplement in patients with known osteoporosis (36.1%), angiotensin-converting enzyme inhibitor (ACEI) for patients with systolic heart failure and/or post myocardial infarction (12.0%), antiplatelet therapy for patients with a documented history of coronary, cerebral or peripheral vascular disease (10.8%), ACEI or angiotensin-receptor blocker for patients with documented proteinuria (9.6%), statin therapy in patients aged < 85 years with a documented history of coronary or cerebral disease (6.0%), beta-blocker for patients with ischaemic heart disease (4.8%), anticoagulant therapy for patients with chronic atrial fibrillation (4.8%), antihypertensive therapy if systolic blood pressure > 140 mmHg and /or diastolic blood pressure > 90 mmHg in patients with diabetes (3.6%), folic acid supplement in patients taking methotrexate (2.4%), and appropriate beta-blocker (bisoprolol, nebivolol, metoprolol or carvedilol) in patients with stable systolic heart failure (2.4%). There were no documented pharmacological or clinical status contraindication existing for the use of these medications.

### Factors independently associated with inappropriate medication use

On the multivariable logistic regression analysis, the findings revealed that one variable was significantly and independently associated with the inappropriate medication use. Respondents taking ≥ 5 medications were found to be using more PIMs according to Beers criteria (OR: 6.3; 95% CI: 3.5–11.3; p<0.001), STOPP criteria (OR: 3.3; 95% CI: 2.0–5.6), FORTA criteria (OR: 6.0; 95% CI: 3.4–10.7), and MAI criteria (OR: 3.9; 95% CI: 2.2–7.0) compared with those taking ≤ 4 medications. Respondents with ≥ 3 chronic diseases were found to have more PPOs according to START criteria (OR: 4.0; 95% CI: 1.8–8.9; p = 0.001) compared with those with 1–2 chronic diseases. Also, PPOs were found to be significantly more common among those with low and intermediate education levels in comparison to those with high education level (p = 0.005). Residence area was significantly and independently associated with inappropriate prescribing according to Beers, FORTA and START criteria (p<0.05). The frequency of inappropriate prescribing based on the Beers criteria was lowest (OR: 0.2; 95% CI: 0.1–0.7; p = 0.001) in Al-Jahra clinics compared with other governorates. According to FORTA criteria, it was highest in Al-Ahmadi clinics (OR:1.8; 95% CI: 1.1–3.1) compared with other governorates. Respondents attending Al-Ahmadi (OR = 0.5; 95% CI: 0.3–0.9) and Hawalli clinic (OR: 0.2; 95% CI: 0.1–0.7) had the lower PPOs in comparison to those attending clinics in the other governorates. Table 4 presents the association between participants with PIMs according to Beers, STOPP, and MAI criteria, and PPOs according to START criteria, and their characteristics.

**Table 4:** Association between participants with PIMs according to Beers, STOPP, FORTA, and MAI criteria, and PPOs according to START criteria, and their characteristics.

Characteristics	Beers Criteria		STOPP Criteria		MAI Criteria		FORTA List		START Criteria	
	OR (95% CI)	p-value	OR (95% CI)	p-value	OR (95% CI)	p-value	OR (95% CI)	p-value	OR (95% CI)	p-value
**Age (Years)**		0.13		-		-		-		0.15
65–74	Reference		-		-		-		Reference	
75–84	1.4 (0.9–2.3)		-		-		-		1.1(0.6–2.0)	
≥ 85	2.5 (0.8–8.1)		-		-		-		0.1(0.02–1.10)	
**Gender**		-		0.06		0.77		0.09		0.13
Male	-		Reference		Reference		Reference		Reference	
Female	-		1.5 (0.9–2.2)		1.1 (0.6–1.8)		1.5 (0.9–2.3)		1.6 (0.9–2.9)	
**Education Level**		-		-		0.81		0.66		0.005*
Low Education	-		-		Reference		Reference		Reference	
Intermediate Education	-		-		0.9 (0.5–1.9)		1.2 (0.7–2.3)		2.0 (1.0–4.1)	
High Education	-		-		0.8 (0.4–1.6)		0.9 (0.4–1.7)		0.4 (0.1–0.9)	
**Residence**		0.001*		-		0.11		0.02*		0.03*
Al-Farwaniyah	Reference		-		Reference		Reference		Reference	
Capital	0.6 (0.3–1.1)		-		0.5 (0.2–1.0)		0.8 (0.4–1.6)		0.9 (0.4–1.9)	
Al-Ahmadi	1.4 (0.8–2.5)		-		1.1 (0.6–2.1)		1.8 (1.1–3.1)		0.5 (0.3–0.9)	
Hawalli	1.9 (0.9–3.9)		-		0.6 (0.3–1.2)		1.6 (0.8–3.2)		0.2 (0.1–0.7.)	
Al-Jahra	0.2 (0.1–0.7)		-		0.7 (0.2–2.3)		0.5 (0.1–1.4)		0.3 (0.1–1.2)	
**Number of Diseases**		0.09		0.61		0.75		0.22		0.001*
1–2	Reference		Reference		Reference		Reference		Reference	
≥ 3	1.6 (0.9–2.7)		1.1 (0.7–1.9)		0.9 (0.6–1.6)		1.4 (0.8–2.4)		4.0 (1.8–8.9)	
**Number of Medications**		<0.001*		<0.001*		<0.001*		<0.001*		0.9
1–4	Reference		Reference		Reference		Reference		Reference	
≥ 5	6.3 (3.5–11.3)		3.3 (2.0–5.6)		6.0 (3.4–10.7)		3.9 (2.2–7.0)		1.0 (0.5–2.1)	

Variables with p > 0.25 in the univariable analysis, which were not included in the multivariable analysis

* P<0.05

### Comparisons and correlations between the explicit and implicit criteria

Table 5 shows the prevalence rates for PIMs and the sensitivity and specificity of the three explicit criteria in comparison to the MAI as reference standard. The MAI was used a reference point due to its reliably and validity as a standardized assessment tool [9, 11]. STOPP had the highest sensitivity (68.6%) to detect PIMs followed by Beers (58.3%), and FORTA (52.4%) criteria. Also STOPP had the highest specificity (80.2%) followed by FORTA (78.4%) and Beers (61.3%) criteria. The measure of agreement (kappa index) was 0.40 between STOPP and MAI criteria, 0.23 between FORTA and MAI criteria, and 0.16 between the Beers and MAI criteria.

**Table 5:** Sensitivity, specificity, and measure of agreement (Kappa).

Variable	MAI	2015 Beers Criteria	STOPP version 2	FORTA
**Prevalence of PIMs (95% CI)**	73.6% (69.0–77.7)	53.1% (48.2–57.9)	55.7% (50.8–60.5)	44.3% (39.5–49.2
**Sensitivity (95% CI)**	Reference	58.3% (52.5–63.8)	68.6% (63.1–73.7)	52.4% (46.7–58.1)
**Specificity (95%CI)**	Reference	61.3% (51.5–70.2)	80.2% (71.3–86.9)	78.4% (69.4–85.4)
**Kappa Index (p-value)**	Reference	0.16 (<0.001)	0.40 (<0.001)	0.23 (<0.001)

Beers and STOPP criteria identified significantly more PIMs compared with FORTA list (p<0.001). There was no significant difference between the number of PIMs identified by Beers and STOPP criteria (p = 0.53). The MAI implicit criteria revealed a significantly higher number of medications with inappropriate ratings compared with the Beers, STOPP and FORTA as explicit criteria (p<0.001). Similarly, the Beers and STOPP criteria identified significantly higher percentages of patients with PIMs compared with FORTA (p = 0.01 and p = 0.001, respectively). There was no significant difference between the percentages of patients with PIMs according to Beers and STOPP criteria (p = 0.49). The MAI identified a significantly higher percentage of patients with inappropriate medications ratings compared with the Beers, STOPP and FORTA criteria (p<0.001). There were significant correlations between PIMs identified by Beers and STOPP criteria (r = 0.238; p<0.001), Beers and MAI criteria (r = 0.172; p<0.001), Beers and FORTA criteria (r = 0.367; p<0.001), STOPP and MAI criteria (r = 0.433; p<0.001), STOPP and FORTA criteria (r = 0.274; p<0.001), and FORTA and MAI criteria (r = 0.273; p<0.001) among the study participants.

## Discussion

The present study appears to be the first literature report, which identified and compared the prevalence of PIMs use among geriatric patients according to four international screening tools applied simultaneously to one collective of patients. PIMs were present in 53.1%, 55.7%, and 44.3% of respondents, according to Beers, STOPP, and FORTA criteria, respectively. Almost 74% of respondents had one or more inappropriate ratings among their medications in the MAI criteria. According to START criteria, 19.8% of patients had at least one PPO. Respondents taking ≥ 5 medications were found to be using significantly more PIMs according to Beers, STOPP, FORTA, and MAI criteria. Beers and STOPP criteria identified significantly more PIMs compared with FORTA. The MAI revealed a significantly higher number of medications with inappropriate ratings compared with the explicit criteria. Taking the MAI as reference standard, STOPP criteria had the highest sensitivity and measure of agreement to detect PIMs in comparison to Beers and FORTA criteria. The present findings determine the baseline prevalence rate of PIMs and PPOs, which would be the first step in evaluating any future interventions to decrease their rate in Kuwaiti primary care settings.

The prevalence rate of PIMs based on 2015 Beers criteria in this study was 53.1%. In comparison to previous studies conducted in similar settings worldwide, this value is within the range (37% to 59.2%) reported in studies that based their evaluation on the 2012 Beers criteria [15, 21, 23, 38, 41]. Moreover, it is higher than the range of 18.3% to 48.0% where the 2003 version of the Beers criteria was used to identify PIMs [14, 15, 18, 20–22, 24, 42]. To the best of our knowledge, no reports exist in literature using the 2015 updated Beers Criteria in primary care settings. However; few studies were published at the inpatient settings [34, 35, 37]. The present findings revealed that 55.7% of patients were prescribed at least one PIM based on STOPP version 2. It is higher than the rates of 21.4% and 35.4% reported in European studies performed in the same healthcare context (primary care) using STOPP version 1 [15, 18]. This could be explained by the fact that 52.6% of PIMs in the present study were related to medications prescribed without an evidence-based clinical indication, medications prescribed beyond the recommended duration, and duplicate therapy, which were not included in STOPP version 1. Forty-four percent of the patients were prescribed PIMs classified as C or D medications according to FORTA criteria, which is lower than the rate reported in hospitalized geriatric patients (58.0%) [33]. Almost three-quarters (73.6%) of the study population had one or more inappropriate ratings among their prescribed medications according to the MAI criteria. It is lower than that reported by previous similar studies performed in Australia (99%) and Denmark (94.3%) [10, 11]. The mean MAI score per medication in the present study (1.0) is similar to that reported by a study conducted in Denmark (1.3) [11]. All comparisons to the previous literature must be considered with caution since multiple possible factors of variability should be taken into account such as, differences in methodologies including, duration of data collection, retrospective or prospective data, health systems including, patient care pathway, prescribing practices, and medications availability.

The inappropriate prescribing practice demonstrated by this study can partially be explained by polypharmacy, which has been identified as a significant predictor of PIMs use among respondents. This finding is in agreement with previously published literature [14–16, 23, 24, 41, 43, 46]. Additional reasons may include lack of comprehensive medicine evaluations for elderly patients, lack of awareness of the primary care physicians regarding the risks of prescribing PIMs, lack of continuous professional education programs addressing this matter, and absence of geriatric specialists. These highlight the need for further research to estimate the level of PIMs awareness among physicians and pharmacists in the primary care settings in Kuwait. Other predictors for inappropriate prescribing reported in the literature were age and gender [5, 14, 22–24], although neither of these two independent factors were significantly associated with inappropriate prescribing in the present study. This finding is consistent with a reported study performed in Spain [15], and a systematic literature review that revealed inconsistent significant association of age and gender with inappropriate prescribing [46]. The current results showed that residence area is significantly associated with inappropriate prescribing according to Beers and FORTA criteria. While the reasons for this regional variation are unclear, it may be due to differences in the physicians’ qualifications, clinical practice, and the health status of the patients. This finding has important implications for future research.

In the present study, the prevalence and pattern of PIMs varied considerably according to the four different screening tools. Taking the MAI as reference standard, STOPP version 2 criteria had the highest sensitivity and measure of agreement to detect PIMs compared to 2015 Beers criteria and 2014 FORTA list. Moreover, the degree of agreement between the 2015 Beers criteria and MAI, and the 2014 FORTA list and MAI was low, which means there was minimal overlap between these criteria. The current result that there was no significant difference between the number of PIMs identified by 2015 Beers and STOPP version 2 criteria is in contrast with two recent studies conducted in Europe that revealed the 2012 Beers criteria to detect the highest number of PIMs [15, 30]. In the present study, 2015 Beers criteria and STOPP version 2 criteria identified significantly more PIMs compared to 2014 FORTA list, which is in consistence with a recent study conducted in Germany, which reported that largest number of PIMs were identified by STOPP compared with FORTA [33]. There was no previous literature that compared the Beers criteria to FORTA list. The MAI implicit criteria revealed a significantly higher number of medications with inappropriate ratings in comparison to the explicit criteria (Beers, STOPP and FORTA), which is in agreement with the previous literature [9, 47]. Both explicit and implicit criteria have their own advantages and limitations. The explicit criteria have limited transferability between various countries due to differences in prescribing practices and they need regular review to keep up to date with evolving clinical evidence. Moreover, a range of limitations were highlighted regarding the applicability and reliability of Beers criteria outside of the United States of America [9, 16, 31]. The MAI was the most time consuming to apply particularly in busy practice and needs a skilled clinician who uses patient-specific information and the available clinical evidence to formulate a clinical judgement relating to the appropriateness or inappropriateness of a specific medication. Furthermore, the MAI score per medication does not assist the physician to prioritize which medications should be changed [9]. The present findings suggest that the explicit criteria can be useful for identification of inappropriate prescribing among elderly patients, particularly the STOPP version 2 that had shown the highest sensitivity and measure of agreement with the MAI compared with the 2015 Beers criteria and 2014 FORTA list. The present findings and the reported limitations of these criteria suggest the need for formulation of a country-specific PIMs list based on the three explicit criteria, which should be tailored and adapted to Kuwaiti realities. It may better reflect the prescribing practices in Kuwait and the medications available in the local pharmaceutical market, and may have important practical applications as guidance to good geriatric care and medication safety practices.

In the present study, 19.8% of patients had one or more clinically indicated medications omitted from their treatment according to START tool without valid reasons, which is close to that reported in a similar setting in Ireland (22.7%) [18]. Higher rates of PPOs were found in the hospital settings of six European countries (59.4%) and Taiwan (41.9%) [17, 26]. A possible explanation for these PPOs may be that some clinicians do not adhere to the clinical guidelines and prefer to treat the patients based on their clinical experiences. Additional probable reasons may be result from the physicians’ desire to avoid polypharmacy due to fear of adverse effects and concerns about non-adherence when prescribing more medications for the patient. However, this study revealed that there was no significant association between polypharmacy and PPOs, which is in agreement with a previous study in Ireland [18]. The current results demonstrated that having ≥ 3 chronic diseases was a significant predictor for PPOs, which may dissuade physicians from adding clearly indicated medicines. This study showed that patients with high education level had significantly lower PPOs compared to those with low and intermediate education levels. This may be due to the fact that participants with high education level had better healthcare information to help make appropriate health decisions. The significant regional variation observed with the START criteria may be due to differences in the physicians’ qualifications, clinical practice, and the health status of the patients. These findings highlight the need for future research to provide better understanding of the education and residency as predictors of PPOs.

Given the harmful effects of PIMs for geriatric patients, future studies are warranted to assess the impact of the inappropriate prescribing demonstrated by this study on clinical outcomes such as medication-related adverse events, hospitalization and mortality among elderly patients in Kuwait. Also, the present findings highlight the need for the implementation of interventional strategies that may improve prescribing pattern in elderly patients [3, 10, 24, 30, 48]. These include (i) continuous professional education for the healthcare providers in the primary care to improve their knowledge and skills in geriatric care; (ii) the development of computer-assisted decision-support systems for electronic prescribing with alerts for PIMs; and (iii) the establishment of an effective multidisciplinary team caring for the elderly patients in which the role of the pharmacists is defined and supported in reviewing, monitoring and optimizing medication therapy in collaboration with the physicians.

### Strengths/Limitations

The strengths of the present study include (i) the use of appropriate sampling method and sample size to generate a representative data about the study population, (ii) the use of data derived from the patients’ medical records through the cooperation of the primary care physicians, which reflected PIMs prescribing more accurately compared with data from pharmacy dispensing records, which are incomplete and do not include any information about the medical history of the patient in Kuwait; (iii) the first study to identify and compare inappropriate prescribing according to four screening tools applied simultaneously to one collective of patients to overcome several limitations of using solely one tool, (iv) and the first study to evaluate prescribing practices for the elderly in primary care settings of Kuwait using the most updated explicit criteria at the time of the study.

There were certain limitations to the current study, which include (i) primary care doctors were not interviewed to identify their reasons for prescribing the identified PIMs and PPOS, and whether they were aware of the prescribing of PIMs or monitoring patients for adverse events with PIMs; (ii) limited generalization of the present findings to the general population, because this study was restricted to the prescription profile of patients at the primary healthcare facilities. Hence, to gain more insight into the medication use among elderly patients in Kuwait, and possible adverse outcomes, further studies are required in the secondary and tertiary healthcare facilities; (iii) the exclusion of OTC medications and herbal supplements may have resulted in an underestimation of PIMs; and (iv) the non-documentation of specific medications in the patient’s medical records, which are prescribed by certain specialists and not available at the primary care settings.

## Conclusions

The current results indicate a considerable amount of inappropriate prescribing among geriatric patients in the primary care settings of Kuwait. Given that the proportion of elderly people is rising, the high prevalence of inappropriate prescribing is a major public health concern. These findings underscore the need for multifaceted interventions that could be targeted at the identified areas to improve the quality of prescribing in elderly patients, to reduce the number of medicines whenever possible, and to increase the appropriateness of the medication regimen. The use of explicit criteria seems to be useful for identification of inappropriate prescribing among elderly patients, particularly the STOPP version 2 that had shown the highest sensitivity and measure of agreement with the MAI compared with the 2015 Beers criteria and 2014 FORTA list.

## Supporting information

S1 Dataset (XLSX)

S2 Dataset (XLSX)

S1 Text (DOCX)

## References

[1] National Institute on Aging. World's older population grows dramatically. [Internet]. 2018 [cited 2018 Jul 9]. Available from: https://www.nia.nih.gov/news/worlds-older-population-grows-dramatically

[2] World Population Review. World Population Prospects (2017 Revision)—United Nations population estimates and projections. [Internet]. 2018 [cited 2018 Jul 4]. Available from: http://worldpopulationreview.com/countries/kuwait-population/

[3] Spinewine A, Schmader KE, Barber N, Hughes C, Lapane KL, Swine C, et al Appropriate prescribing in elderly people: how well can it be measured and optimised? Lancet. 2007;370:173–184. 10.1016/S0140-6736(07)61091-5 17630041

[4] Fu AZ, Jiang JZ, Reeves JH, Fincham JE, Liu GG, Perri M. Potentially inappropriate medication use and healthcare expenditures in the US community-dwelling elderly. Med Care. 2007;45:472–476. 10.1097/01.mlr.0000254571.05722.34 17446834

[5] Morgan SG, Hunt J, Rioux J, Proulx J, Weymann D, Tannenbaum C. Frequency and cost of potentially inappropriate prescribing for older adults: a cross-sectional study. CMAJ Open. 2016;4:E346–351. 10.9778/cmajo.20150131 27398383 PMC4933607

[6] Fick DM, Semla TP, Beizer J, Brandt N, Dombrowski R, DuBeau CE, et al American Geriatrics Society 2015 Updated Beers Criteria for Potentially Inappropriate Medication Use in Older Adults. J Am Geriatr Soc. 2015;63:2227–2246. 10.1111/jgs.13702 26446832

[7] O’Mahony D, O’Sullivan D, Byrne S, O’Connor MN, Ryan C, Gallagher P. STOPP/START criteria for potentially inappropriate prescribing in older people: version 2. Age Ageing. 2015;44:213–218. 10.1093/ageing/afu145 25324330 PMC4339726

[8] Pazan F, Weiss C, Wehling M. The EURO-FORTA (Fit fOR The Aged) List: International Consensus Validation of a Clinical Tool for Improved Drug Treatment in Older People. Drugs Aging. 2018;35:61–71. 10.1007/s40266-017-0514-2 29335932

[9] Hanlon JT, Schmader KE. The medication appropriateness index at 20: where it started, where it has been, and where it may be going. Drugs Aging. 2013;30:893–900. 10.1007/s40266-013-0118-4 24062215 PMC3831621

[10] Castelino RL, Bajorek BV, Chen TF. Retrospective evaluation of home medicines review by pharmacists in older Australian patients using the medication appropriateness index. Ann Pharmacother. 2010;44:1922–1929. 10.1345/aph.1P373 21119095

[11] Bregnhøj L, Thirstrup S, Kristensen MB, Bjerrum L, Sonne J. Prevalence of inappropriate prescribing in primary care. Pharm World Sci. 2007;29:109–115. 10.1007/s11096-007-9108-0 17353970

[12] Michalek C, Wehling M, Schlitzer J, Frohnhofen H. Effects of “Fit fOR The Aged” (FORTA) on pharmacotherapy and clinical endpoints-a pilot randomized controlled study. Eur J Clin Pharmacol. 2014; 70: 1261–1267. 10.1007/s00228-014-1731-9 25128076

[13] Somers A, Robays H, De Paepe P, Van Maele G, Perehudoff K, Petrovic M. Evaluation of clinical pharmacist recommendations in the geriatric ward of a Belgian university hospital. Clin Interv Aging. 2013;8:703–709. 10.2147/CIA.S42162 23807844 PMC3686245

[14] Buck MD, Atreja A, Brunker CP, Jain A, Suh TT, Palmer RM, et al Potentially inappropriate medication prescribing in outpatient practices: prevalence and patient characteristics based on electronic health records. Am J Geriatr Pharmacother. 2009;7:84–92. 10.1016/j.amjopharm.2009.03.001 19447361

[15] Blanco-Reina E, Ariza-Zafra G, Ocan˜a-Riola R, Leo´n-Ortiz M. 2012 American Geriatrics Society Beers criteria: enhanced applicability for detecting potentially inappropriate medications in European older adults? A comparison with the screening tool of older person’s potentially inappropriate prescriptions. J Am Geriatr Soc. 2014;62:1217–1223. 10.1111/jgs.12891 24917083

[16] O’Sullivan DP, O’Mahony D, Parsons C, Hughes C, Murphy K, Patterson S, et al A prevalence study of potentially inappropriate prescribing in Irish long-term care residents. Drugs Aging 2013;30:39–49. 10.1007/s40266-012-0039-7 23229766

[17] Gallagher P, Lang PO, Cherubini A, Topinková E, Cruz-Jentoft A, Montero Errasquín B, et al Prevalence of potentially inappropriate prescribing in an acutely ill population of older patients admitted to six European hospitals. Eur J Clin Pharmacol. 2011;67:1175–1188. 10.1007/s00228-011-1061-0 21584788

[18] Ryan C, O’Mahony D, Kennedy J, Weedle P, Byrne S. Potentially inappropriate prescribing in an Irish elderly population in primary care. Br J Clin Pharmacol. 2009;68:936–947. 10.1111/j.1365-2125.2009.03531.x 20002089 PMC2810806

[19] Gallagher P, O’Mahony D. STOPP (Screening Tool of Older Persons’ potentially inappropriate Prescriptions): application to acutely ill elderly patients and comparison with Beers’ criteria. Age Ageing. 2008;37:673–679. 10.1093/ageing/afn197 18829684

[20] de Oliveira Martins S, Soares MA, Foppe van Mil JW, Cabrita J. Inappropriate drug use by Portuguese elderly outpatients—effect of the Beers criteria update. Pharm World Sci. 2006;28:296–301. 10.1007/s11096-006-9046-2 17111245

[21] Baldoni Ade O, Ayres LR, Martinez EZ, Dewulf Nde L, Dos Santos V, Pereira LR. Factors associated with potentially inappropriate medications use by the elderly according to Beers criteria 2003 and 2012. Int J Clin Pharm. 2014;36:316–324. 10.1007/s11096-013-9880-y 24271923

[22] Faustino CG, Passarelli MC, Jacob-Filho W. Potentially inappropriate medications among elderly Brazilian outpatients. Sao Paulo Med J. 2013;131:19–26. 23538591 10.1590/S1516-31802013000100004 PMC10852081

[23] Nishtala PS, Bagge ML, Campbell AJ, Tordoff JM. Potentially inappropriate medicines in a cohort of community-dwelling older people in New Zealand. Geriatr Gerontol Int. 2014;14:89–93. 10.1111/ggi.12059 23530567

[24] Lai HY, Hwang SJ, Chen YC, Chen TJ, Lin MH, Chen LK. Prevalence of the prescribing of potentially inappropriate medications at ambulatory care visits by elderly patients covered by the Taiwanese National Health Insurance program. Clin Ther. 2009;31:1859–1870. 10.1016/j.clinthera.2009.08.023 19808145

[25] Chen LL, Tangiisuran B, Shafie AA, Hassali MA. Evaluation of potentially inappropriate medications among older residents of Malaysian nursing homes. Int J Clin Pharm. 2012;34:596–603. 10.1007/s11096-012-9651-1 22622593

[26] Liu CL, Peng LN, Chen YT, Lin MH, Liu LK, Chen LK. Potentially inappropriate prescribing (IP) for elderly medical inpatients in Taiwan: a hospital-based study. Arch Gerontol Geriatr. 2012;55:148–1451. 10.1016/j.archger.2011.07.001 21820189

[27] Ubeda A, Ferrándiz L, Maicas N, Gomez C, Bonet M, Peris JE. Potentially inappropriate prescribing in institutionalised older patients in Spain: the STOPP-START criteria compared with the Beers criteria. Pharm Pract (Granada). 2012;10:83–91. 24155822 10.4321/s1886-36552012000200004 PMC3780483

[28] Conejos Miquel MD, Sánchez Cuervo M, Delgado Silveira E, Sevilla Machuca I, González-Blazquez S, Montero Errasquin B, et al Potentially inappropriate drug prescription in older subjects across health care settings. Eur Geriatr Med. 2010;1:9–14.

[29] O’Mahony D, Gallagher P, Ryan C, Byrne S, Hamilton H, Barry P, et al STOPP & START criteria: a new approach to detecting potentially inappropriate prescribing in old age. Eur Geriatr Med. 2010;1:45–51.

[30] Di Giorgio C, Provenzani A, Polidori P. Potentially inappropriate drug prescribing in elderly hospitalized patients: an analysis and comparison of explicit criteria. Int J Clin Pharm. 2016;38:462–468. 10.1007/s11096-016-0284-7 26984238

[31] Hamilton H, Gallagher P, Ryan C, Byrne S, O'Mahony D. Potentially inappropriate medications defined by STOPP criteria and the risk of adverse drug events in older hospitalized patients. Arch Intern Med. 2011;171:1013–1019. 10.1001/archinternmed.2011.215 21670370

[32] Mucalo I, Hadžiabdić MO, Brajković A, Lukić S, Marić P, Marinović I, et al Potentially inappropriate medicines in elderly hospitalised patients according to the EU(7)-PIM list, STOPP version 2 criteria and comprehensive protocol. Eur J Clin Pharmacol. 2017;73:991–999. 10.1007/s00228-017-2246-y 28405697

[33] Wickop B, Härterich S, Sommer C, Daubmann A, Baehr M, Langebrake C. Potentially Inappropriate Medication Use in Multimorbid Elderly Inpatients: Differences Between the FORTA, PRISCUS and STOPP Ratings. Drugs Real World Outcomes. 2016;3:317–325 10.1007/s40801-016-0085-2 27747830 PMC5042941

[34] Sheikh-Taha M, Dimassi H. Potentially inappropriate home medications among older patients with cardiovascular disease admitted to a cardiology service in USA. BMC Cardiovasc Disord. 2017; 17:189 10.1186/s12872-017-0623-1 28716041 PMC5514488

[35] Zhang X, Zhou S, Pan K, Li X, Zhao X, Zhou Y, et al Potentially inappropriate medications in hospitalized older patients: a cross-sectional study using the Beers 2015 criteria versus the 2012 criteria. Clin Interv Aging. 2017; 12:1697–1703. 10.2147/CIA.S146009 29066875 PMC5644572

[36] Grina D, Briedis V. The use of potentially inappropriate medications among the Lithuanian elderly according to Beers and EU(7)-PIM list—a nationwide cross-sectional study on reimbursement claims data. J Clin Pharm Ther. 42:195–200. 10.1111/jcpt.12494 28155237

[37] Komagamine J. Prevalence of potentially inappropriate medications at admission and discharge among hospitalised elderly patients with acute medical illness at a single centre in Japan: a retrospective cross-sectional study. BMJ Open. 2018; 8:e021152 10.1136/bmjopen-2017-021152 30030316 PMC6059264

[38] Al Odhayani A, Tourkmani A, Alshehri M, Alqahtani H, Mishriky A. Potentially inappropriate medications prescribed for elderly patients through family physicians. Saudi J Biol Sci. 2017;24:200–2007. 10.1016/j.sjbs.2016.05.006 28053591 PMC5198987

[39] Bahlas SM, Anshasi NW, Alqurashi SH, Elhosiny AA, Kamal EK, Andijani RA. Evaluating Potentially Inappropriate Medications in the Elderly Population at KAUH, Jeddah, KSA. Int J Adv Res. 2017; 5:540–545.

[40] Al-Omar HA, Al-Sultan MS, Abu-Auda HS. Prescribing of potentially inappropriate medications among the elderly population in an ambulatory care setting in a Saudi military hospital: trend and cost. Geriatr Gerontol Int. 2013;13:616–621. 10.1111/j.1447-0594.2012.00951.x 23035714

[41] Zeenny R, Wakim S, Kuyumjian YM. Potentially inappropriate medications use in community-based aged patients: a cross-sectional study using 2012 Beers criteria. Clin Interv Aging. 2017;12:65–73. 10.2147/CIA.S87564 28115835 PMC5221543

[42] Saab YB, Hachem A, Sinno S, El-Moalem H. Inappropriate medication use in elderly Lebanese outpatients: prevalence and risk factors. Drugs Aging. 2006;23:743–752. 10.2165/00002512-200623090-00004 17020398

[43] Alhmoud E, Khalifa S, Bahi AA. Prevalence and predictors of potentially inappropriate medications among home care elderly patients in Qatar. Int J Clin Pharm. 2015;37:815–821. 10.1007/s11096-015-0125-0 25986290

[44] Raosoft. Sample Size Calculator. [Internet]. 2018 [cited 2018 Jul 27]. Available from: http://www.raosoft.com/samplesize.htmlCalculator.

[45] Kuhn-Thiel AM, Weiß C, Wehling M. FORTA authors/expert panel members. Consensus validation of the FORTA (Fit fOR The Aged) List: a clinical tool for increasing the appropriateness of pharmacotherapy in the elderly. Drugs Aging. 2014;31:131–140. 10.1007/s40266-013-0146-0 24353033 PMC3907693

[46] Tommelein E, Mehuys E, Petrovic M, Somers A, Colin P, Boussery K. Potentially inappropriate prescribing in community-dwelling older people across Europe: a systematic literature review. Eur J Clin Pharmacol. 2015;71:1415–1427. 10.1007/s00228-015-1954-4 26407687

[47] Steinman MA, Rosenthal GE, Landefeld CS, Bertenthal D, Sen S, Kaboli PJ. Conflicts and concordance between measures of medication prescribing quality. Med Care. 2007;45:95–99. 10.1097/01.mlr.0000241111.11991.62 17279026

[48] Wawruch M, Zikavska M, Wsolova L, Jezova D, Fialova D, Kunzo M, et al Perception of potentially inappropriate medication in elderly patients by Slovak physicians. Pharmacoepidemiol Drug Saf. 2006;15:829–834. 10.1002/pds.1290 16927435

