# Supplementary material for: Potentially inappropriate medication use among geriatric patients in primary care setting: A cross-sectional study using the Beers, STOPP, FORTA and MAI criteria
Source: PLoS One. 2019 Jun 13;14(6):e0218174. doi: 10.1371/journal.pone.0218174 (PMC6563997; doi:10.1371/journal.pone.0218174)
Supplement: S1 Text — (DOCX) [file pone.0218174.s003.docx]

**Evaluation of Medication Use in Elderly Patients**

Name of Polyclinic ………………………………….. Governorate………………

Patient ID……………………………………………..

**Section A: Demographic Characteristics**

1. **Age** (in years) ……………
2. **Gender**  Male Female
3. **Education level** Less than high school High school Diploma University

Postgraduate

1. **Residence**  Capital Hawalli Al-Farwaniya Al-Ahmadi Al-Jahra

1. **Smoking habits**

Never smoked
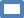
 Smoking…… cigarettes/day for …. years
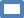
 Stopped ……… year(s) ago
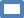


**Section B: Chronic Diseases and Medications**

1. **Chronic Diseases**

| Hypertension | Yes | No | Chronic Kidney Disease (if yes, Stage) | Yes | No |
| --- | --- | --- | --- | --- | --- |
| Ischemic Heart Disease | Yes | No | Rheumatoid arthritis | Yes | No |
| Chronic Heart Failure | Yes | No | Osteoarthritis | Yes | No |
| Dyslipidemia | Yes | No | Osteoporosis | Yes | No |
| Previous Stroke/TIA | Yes | No | Depression | Yes | No |
| Diabetes | Yes | No | Parkinson’s Disease | Yes | No |
| Asthma | Yes | No | Dementia | Yes | No |
| Chronic Obstructive Pulmonary Disease | Yes | No | Urinary incontinence |  |  |
| Others (Specify) |  |  |  |  |  |

1. **Prescribed Medications**

| **Name of the Medicine/ Strength** | **Dosage Form** | **Dose Schedule/**  **Frequency** | **Direction of use** | **Duration of use** |
| --- | --- | --- | --- | --- |
|  |  |  |  |  |
|  |  |  |  |  |
|  |  |  |  |  |
|  |  |  |  |  |
|  |  |  |  |  |
|  |  |  |  |  |
|  |  |  |  |  |
|  |  |  |  |  |
|  |  |  |  |  |
|  |  |  |  |  |
|  |  |  |  |  |
|  |  |  |  |  |
|  |  |  |  |  |
